# Recent Research for Unobtrusive Atrial Fibrillation Detection Methods Based on Cardiac Dynamics Signals: A Survey

**DOI:** 10.3390/s21113814

**Published:** 2021-05-31

**Authors:** Fangfang Jiang, Yihan Zhou, Tianyi Ling, Yanbing Zhang, Ziyu Zhu

**Affiliations:** College of Medicine and Biological Information Engineering, Northeastern University, Shenyang 110169, China; 20185423@stu.neu.edu.cn (Y.Z.); 20185538@stu.neu.edu.cn (T.L.); 20185527@stu.neu.edu.cn (Y.Z.); 20185475@stu.neu.edu.cn (Z.Z.)

**Keywords:** atrial fibrillation, unobtrusive detection, ballistocardiogram, seismocardiogram, photoplethysmogram

## Abstract

Atrial fibrillation (AF) is the most common cardiac arrhythmia. It tends to cause multiple cardiac conditions, such as cerebral artery blockage, stroke, and heart failure. The morbidity and mortality of AF have been progressively increasing over the past few decades, which has raised widespread concern about unobtrusive AF detection in routine life. The up-to-date non-invasive AF detection methods include electrocardiogram (ECG) signals and cardiac dynamics signals, such as the ballistocardiogram (BCG) signal, the seismocardiogram (SCG) signal and the photoplethysmogram (PPG) signal. Cardiac dynamics signals can be collected by cushions, mattresses, fabrics, or even cameras, which is more suitable for long-term monitoring. Therefore, methods for AF detection by cardiac dynamics signals bring about extensive attention for recent research. This paper reviews the current unobtrusive AF detection methods based on the three cardiac dynamics signals, summarized as data acquisition and preprocessing, feature extraction and selection, classification and diagnosis. In addition, the drawbacks and limitations of the existing methods are analyzed, and the challenges in future work are discussed.

## 1. Introduction

Atrial fibrillation (AF) is one of the most common arrhythmias that increases the risk of heart diseases, such as cardiogenic stroke and heart failure. According to the World Heart Federation, AF has become a global health problem as morbidity and mortality have increased exponentially over the past decade [1]. With global aging, it is predicted that AF may affect 12 million people in the United States by 2050 and 17.9 million people in Europe by 2060 [2]. Therefore, precise AF detection is crucial for early diagnosis and treatment of AF and even more serious heart diseases.

AF is derived from a chaotic, high-frequency heartbeat, a type of disorganized activity caused by atria [3]. Generally, AF can be categorized as paroxysmal, persistent, and permanent based on the fibrillation duration. Paroxysmal AF is usually asymptomatic, difficult to detect in daily life, and may deteriorate to persistent and permanent or even cause various malignant cardiovascular diseases [4]. Hence, numerous research directions on AF detection have been proposed and implemented to diagnose AF in clinical and daily life.

Currently, the gold standard for AF detection is the electrocardiogram (ECG) signal, according to irregular rhythms lasting for more than 30 s and the disappearance of the P wave in front of the QRS complex [5]. The measurement and acquisition of traditional ECG signals require electrodes adhered to the body surface, professional equipment, and operators suitable for clinical diagnosis. However, AF, especially paroxysmal AF, usually occurs in daily life without obvious symptoms, so various non-invasive measurement techniques have been proposed to monitor AF at home in recent years. The up-to-date unobtrusive AF detecting methods include ECG signals from portable devices and cardiac dynamics signals. In cardiac dynamics signals, cardiac activity is considered as a nonlinear dynamic system, and the response on the body surface reflects changes in cardiac force and rhythm. Therefore, the cardiac dynamics signal has the same rhythm as the ECG signal, which helps diagnose arrhythmias, especially in the detection of AF. In addition, cardiac dynamics signals are generally collected by mechanical sensors to record subtle vibrations on the body surface and analyze the signal waveforms to obtain cardiovascular characterizations. Due to its unobtrusiveness and convenience, it has been widely studied in the field of routine AF detection and screening over the past few years. The state-of-the-art unobtrusive cardiac dynamics methods used for diagnosing AF include ballistocardiogram (BCG), seismocardiogram (SCG) and photoplethysmogram (PPG). The purpose of this paper is to provide a comprehensive summary of previous work on unobtrusive AF detection methods based on the three cardiac dynamics signals, which could aid in developing future research and guidance of ideas for home AF monitoring.

**Figure 1:**
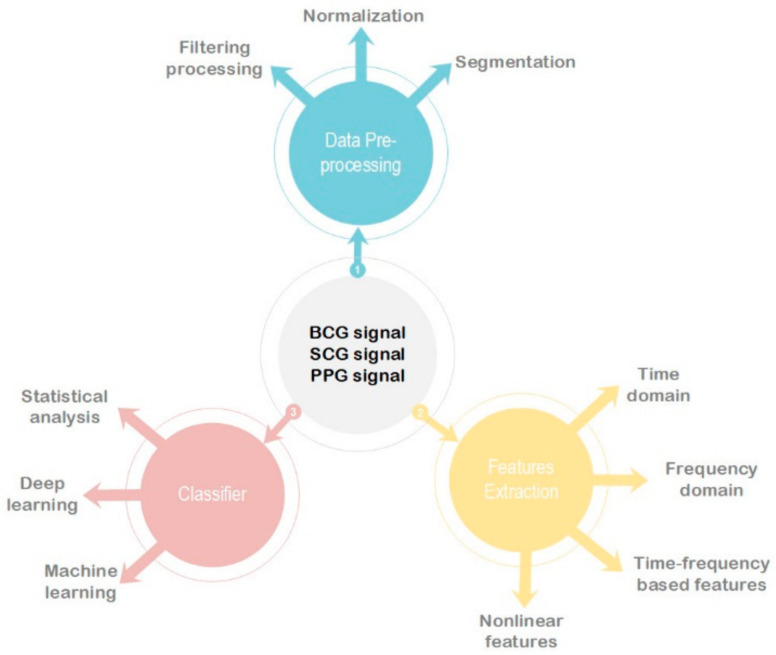
Overview of the cardiac dynamics signals processing for AF detection.

The main detection processes of these three methods commonly include: signal acquisition and preprocessing, feature extraction and selection, classification and diagnosis, as illustrated in Figure 1. Therefore, the rest of the paper is organized as follows: Section 2 focuses on data acquisition equipment and preprocessing methods. Section 3 and Section 4 summarize the feature extraction methods and classification models, respectively. Finally, Section 5 presents the advantages and disadvantages of previous methods and future work in AF detection.

## 2. Instrument/Signal

### 2.1. BCG Signal

**Figure 2:**
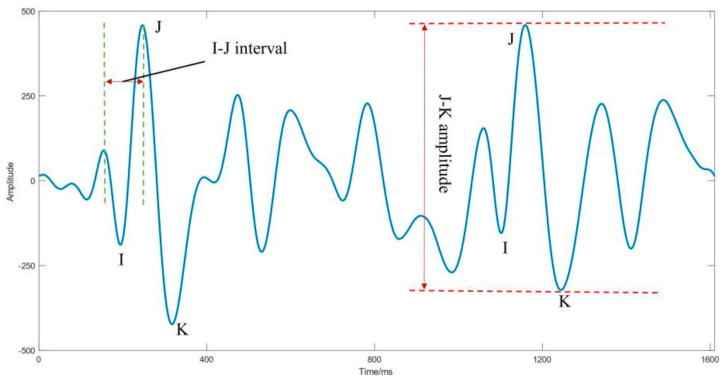
Typical BCG waveform.

BCG signal is an unobtrusive measurement that records subtle vibrations on the body surface to describe cardiac diastolic and systolic forces [6]. The BCG signal has a rhythm similar to the ECG signal, so arrhythmias, including AF, can be detected. Similar to the ECG signal, the morphology of cardiac dynamics signals represents the corresponding cardiovascular events. Figure 2 illustrates the typical morphology of the BCG signal.

The time interval between the peaks of the first major wave (called the I wave) and the second major wave (called the J wave) is called the I-J interval, which represents diastolic pressure. Moreover, the amplitude between the J wave and the third major wave (called the K wave) is called the J-K amplitude and represents pulse pressure [7]. Due to the convenience of data acquisition and almost no negative impact on daily activities, BCG signals have been consistently applied to assess cardiac function in recent years [8]. The main postures during data collection include standing, sitting, and lying down. Moreover, common sensors include wearable three-axis accelerometer [9,10,11], electromechanical film (EMFi) sensor [12], weighing scale [13], piezoelectric film sensors [14], and polyvinylidene fluoride sensors (PVDF) [15].

As far as AF detection is concerned, EMFi and PVDF sensors are installed on cushions and bed mattresses to collect BCG signals. Wen et al. placed the EMFi sensor under a common mattress with a sampling rate of 125 Hz to collect BCG signals and used it to classify AF and sinus rhythm (SR) [16]. Yu et al. placed the EMFi sensor on the top of a regular bed mattress with a sampling rate of 125 Hz to collect BCG signals and detected AF [2]. Brüser et al. and Zink et al. used an EMFi sensor with a sampling rate of 128 Hz to collect BCG signals and detected AF, respectively [17,18,19]. Jiang et al. designed an acquisition system composed of a PVDF sensor with a sampling rate of 125 Hz and placed it on the top of the mattress. The obtained BCG signal corresponded to the upper part of the back of the patients’ body and was used to distinguish AF from non-atrial fibrillation (NAF) [20,21]. Koivisto et al. also applied the Murata EMFi sensor with a sampling rate of 125 Hz to collect BCG signals and detected AF from SR [22]. Moreover, Panula et al. collected BCG signals with a three-axis accelerometer and a three-axis gyroscope to detect AF [23].

### 2.2. SCG Signal

**Figure 3:**
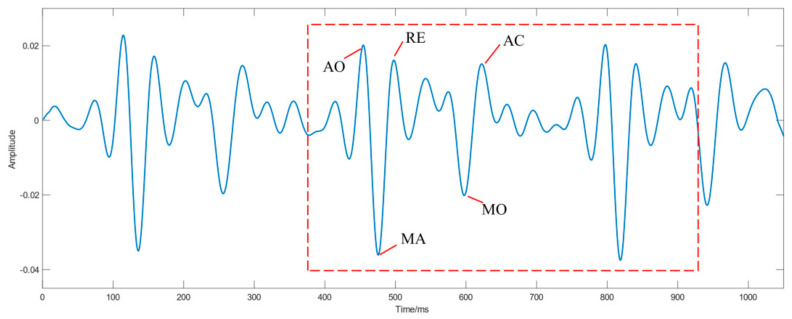
Typical SCG waveform.

SCG signal is a non-invasive method based on measuring chest cardiogenic acceleration, which is a reflection of the heartbeat in the form of local vibrations of the chest wall [6]. SCG is a dynamic manifestation of electrophysiology with a similar rhythm to ECG, thus having the potential to differentiate AF. Figure 3 illustrates the typical morphology of the SCG signal.

There are five important feature points in the SCG waveform: aortic opening (AO), rapid ejection (RE), maximum acceleration (MA), mitral opening (MO) and aortic closure (AC) [24]. Currently, it is convenient to collect SCG signals by placing a low-noise accelerometer on the chest [8]. Moreover, the main sensors for SCG signal detection include: three-axis microelectromechanical sensor (MEMS) accelerometer [25], single-axis MEMS accelerometer [26], MagIC-SCG device with three-axis MEMS accelerometer [27], and electromechanical film (EMFi) sensor [28,29].

As far as AF detection is concerned, MEMS sensors are most commonly used to record SCG signals, which have the advantages of low volume, low power consumption, and low noise. Currently, there are two types of MEMS applied.

One is using a stand-alone MEMS to collect SCG signals for AF detection. Koivisto et al. applied a three-axis MEMS accelerometer fixed in the center of the chest with double-sided glue to collect SCG signals with a sampling rate of 800 Hz, which was used to detect AF and SR [30]. Hurnanen et al. implemented a similar work to record SCG signals with a sampling rate of 800 Hz, ensuring sufficient temporal resolution for classification between AF and SR [31]. Pänkäälä et al. used the single-axis MEMS accelerometers to obtain SCG signals with a sampling rate of 3 kHz for detecting asymptomatic AF [24]. Kaisti et al. applied a MEMS pressure sensor element with a sampling rate of 1 kHz to collect SCG signals and detect AF [32].

The other is the micro MEMS integrated into the mobile phone to monitor AF with SCG. Moreover, the sampling rate is usually set to 200 Hz. Lahdenoja et al. placed a standard Sony Xperia Z-Series smartphone on the patient’s chest with the smartphone’s speaker facing the patient’s head and the display upward to collect the SCG signal and classify AF and NAF [33]. Mehrang et al. and Tadi et al. severally placed a smartphone on the patient’s chest longitudinally to collect SCG signals for distinguishing AF from SR [4,34]. Iftikhar et al. placed a smartphone on the patient’s sternum and classified multiple heart conditions, such as AF, SR, coronary artery disease (CAD), and ST-segment elevated myocardial infarction (STEMI) by SCG signals [35]. Mehrang et al. placed a smartphone on the patient’s bare chest and detected concurrent AF and acute decompensated HF (ADHF) based on the SCG signals collected from the smartphone [36].

### 2.3. PPG Signal

**Figure 4:**
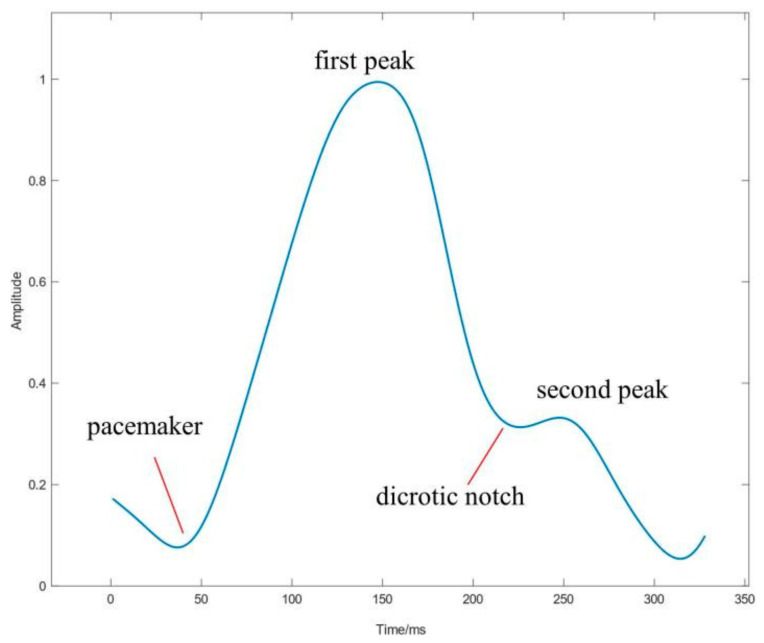
Typical PPG waveform.

PPG signal is a type of pulse pressure signal generated by the propagation of blood pressure pulses along arteries [37]. It can obtain oxygen saturation and heart rate by pulse oximetry, which is practical for detecting AF. Figure 4 illustrates the typical morphology of PPG signals.

The pacemaker represents the opening of the aortic valve and the rapid ejection of blood from the ventricles; the first peak is the highest point of the PPG wave, reflecting the maximum pressure and volume in the artery; the dicrotic notch is the depression of the main and secondary peaks, which represents the static pressure emptiness time of the aorta; the second peak is the transmission of the blood pressure wave from the blood vessel to the end of the body, which bounces back and causes a temporary dilation of the blood vessel wall at the measuring end [38]. It can be measured from fingers, wrists, or earlobes [39] or even by noncontact methods, such as cameras [40].

As far as AF detection is concerned, two main approaches are used to obtain PPG signals: photodetectors (PD) and cameras [37]. For PD-based PPG sensors, Bonomi et al. and Eerikäinen et al. severally used a wrist wearable sensor like a smartwatch with a sampling rate of 128 Hz to collect PPG signals to classify AF and NAF [41,42,43]. Barshar et al. also used the PD in a smartwatch to collect a PPG signal and detect AF from SR [44]. Shashikumar et al. applied a novel deep neural network approach with a sampling rate of 128 Hz to classify AF and NAF from wrist-worn PPG signals [45]. Conroy et al. used a single earlobe PPG sensor with a sampling rate of 300 Hz to record PPG signals and detect the AF and NAF [46]. Aliamiri et al. used a Samsung gear device with a sampling rate of 100 Hz for collecting PPG signals and detected AF [47]. Tang et al. set up a standard procedure to collect fingertip PPG analog data directly from the bedside monitor with a sampling rate of 128 Hz to distinguish the AF and NAF [48]. Ferranti et al. applied the PD in a wristband with a sampling rate of 64 Hz to collect PPG signals and detect AF [49].

For Camera-based PPG sensors, Couderc et al. used an RGB webcam with a sampling rate of 200 Hz, which was placed 1 m above the patient’s head so that the PPG signal could be recorded to detect AF [50]. Moreover, Poh et al. Chan et al. and Krivoshei et al. used a smartphone with a sampling rate of 30 Hz, where the device was placed on the tip of the index finger to extract the PPG signal for distinguishing AF and SR [51,52,53]. Gallego et al. collected the PPG signals with the sensor embedded in the Samsung smartphone to detect AF [54].

To sum up, the cardiac dynamics signals acquisition methods for AF detection are listed in Table 1.

### 2.4. Data Preprocessing

The cardiac dynamics signals preprocessing methods for AF detection comprise filtering, normalization and segmentation.

For filtering processing, various filters with different cutoff frequencies are applied to obtain pure cardiac dynamics signals. In terms of SCG signal, fast Fourier-transform (FFT) based brick-wall filters are effective, with passband frequencies typically set from 1 Hz to 45 Hz to remove baseline wander and high-frequency noise from SCG signals [9,31,35,36]. Moreover, sliding window root-mean-square (RMS) filters with passband frequency of 1 Hz to 40 Hz have also been applied to denoise SCG signal [16,31,33]. Additionally, a triangular window moving-average filter with a length of 8 sampling points was designed to filter SCG signals [34]. In terms of BCG signals, Yu et al. designed a Butterworth band-pass filter with a passband frequency of 0.7 Hz to 10 Hz to remove the noise and respiratory components from BCG signals [2]. Panula et al. applied an equiripple finite impulse response (FIR) band-pass filter of 148th order with passband frequencies ranging from 3 Hz to 30 Hz to denoise BCG signals [23]. In terms of PPG signal, Bashar et al. filtered the PPG signal using a 6th order Butterworth band-pass filter with passband frequencies ranging from 0.5 to 20 Hz [44]. Estrella-Gallego et al. utilized an exponentially weighted moving average (EWMA) filter to denoise PPG signals [54]. Shashikumar et al. applied a 41st order FIR band-pass filter with passband frequencies from 0.2 to 10 Hz to filter PPG signals [45].

In addition, the time-frequency transform is successfully applied to filter cardiac dynamics signals. For BCG, the Daubechies 6 wavelet was applied to decompose the original signal into seven wavelets (D1–D7), and the filtered signal was reconstructed by D3–D6 [16]. Similarly, the time-invariant stationary wavelet transform (SWT) can also be applied to extract relevant features from each of six detail and six coarse coefficients [2]. For SCG, the singular spectrum analysis (SSA) time-series analysis method was used to remove the noise and the de-baseline trend and further smooth the SCG signal derived from accelerometers and gyroscopes [34]. For PPG, Daubechies wavelet was utilized to decompose the original signal into 8-layer wavelets and reconstructed as filters [47].

For normalization, the aim is to eliminate the effects of different devices and subjects. However, its effectiveness and necessity are controversial, so few studies have employed normalization in preprocessing. Wen et al. normalized the amplitude of BCG signals to 0 to 1 by the maximum. Based on empirical values, the lower upper thresholds of the valid signal were set to 0.1 and 0.25 [16]. Yu et al. also normalized a BCG signal with its maximum and minimum value [2]. Conroy et al. normalized the PPG signals using the standard score method, called the Z score [46,55,56].

For the segmentation, different frame lengths were selected, which could describe the rhythmical changes in AF periods. The frame length of BCG signal was usually set as 5 s [22], 24 s [20,21], 30 s [2,17,18] or 60 s [16]. The frame length of SCG signal was generally set as 5 s [22,23], 10 s [4,33,34,35,36] or 12.5 s [30,31], 30 s [41,45]. Moreover, the frame length of PPG signal was commonly set as 1.66 s [46], 5 s [32], 10 s [57], 15 s [50], 17 s [51], 17.1 s [52], 30 s [40,43,44,45,47,54], or 60 s [42].

## 3. Features Extraction

After acquiring the preprocessed data segments, feature extraction is a key link in SR and AF classification. The characterization performance of the extracted features fundamentally determines the accuracy of the ultimate classification and diagnosis. For AF detection, the most used features extracted from cardiac dynamics signals can be classified into the following categories: time-domain, frequency-domain, time-frequency-domain and nonlinear features.

### 3.1. Time-Domain Features

Time-domain features describe the variation of cardiac force over time, a statistical method to describe time-series, such as peaks and troughs, means, and standard deviations [58]. Similar to the features extracted to diagnose AF using ECG signals, the different morphological features and rhythmic features between SR and AF are also represented in the cardiac dynamics signals.

(a) Signal morphology: Similar to ECG signals in AF, such as the absence of P waves, the cardiac dynamics signals also have a morphology that distinguishes AF from SR [59]. For AF diagnosis, Brüser et al. calculated the skewness and kurtosis of the BCG segment using the *k*th sample moments around the mean [17]. Two years later, four new waveform features were supplemented, including the skewness, the kurtosis, the standard deviation, the difference between the maximum and minimum values of each segment [18]. Pänkäälä et al. extracted the variance of the difference between maximum and minimum to describe SCG waveforms [24]. Tadi et al. extracted the zero-crossing ratio of SCG waveforms to detect AF [34]. Additionally, the entire SCG, PPG or BCG segment was fed into a specific classifier to diagnose AF automatically [20,30,47,51].

(b) Time interval: Time interval refers to the interval between two feature points (peaks or troughs) in the time domain waveform. For AF detection, Pänkäälä et al. extracted the AO interval from SCG signals to characterize the variational rhythms [24]. Bonomi et al. calculated the beat-to-beat intervals (BBI) of PPG signals to distinguish AF from NAF [41,42].

(c) Heart rate: Due to the arrhythmia of AF, the heart rate (HR) is usually used as the primary diagnostic indicator. For AF diagnosis, Tadi et al. and Iftikhar et al. applied short-term autocorrelation of SCG segments to obtain the instantaneous heart rate (IHR), respectively [34,35]. Mehrang et al. also adopted short-term autocorrelation from SCG signals to estimate HR [36]. Lahdenoja et al. calculated HR from the median of eight BBI from each SCG segment to identify AF [33]. A. Estrella-Gallego et al. extracted HR using the location of each PPG trough to detect AF [54].

(d) Heart rate variability: Heart rate variability (HRV) refers to the new parameter extracted from the obtained BBI series mathematically. For instance, the root-mean-square of the successive difference (RMSSD) [34,40,44,48,53,58,59] and the standard deviation (SD) [40,43,45,58,59] are commonly used as HRV indicators to characterize AF. In addition, Shashikumar et al. calculated a robust version of SD and a weighted SD feature from the PPG BBI series to describe the rhythm variation [45]. Shi et al. and Conroy et al. severally extracted four HRV features from PPG BBI based on RMMSD and SD to quantify the irregularity of HR [40,46]. Moreover, the median absolute difference and its related parameters were calculated to estimate the HRV of the SCG BBI series [31,33,34,35]. Song et al. used the mean and SD of the BCG BBI series to distinguish AF [58].

### 3.2. Frequency-Domain Features

Frequency domain analysis is an effective and commonly used method for describing rhythmic variations. Generally, the signal is transformed into the frequency domain by FFT, and the frequency features are extracted from the frequency spectrum.

For AF diagnosis, Shan et al. applied FFT to extract three features of the PPG signal, including the low-frequency (LF), high-frequency (HF) and the ratio of LF to HF (LF/HF) [48,59]. The same frequency features of PPG signals were calculated by Shi et al., and the power in LF/HF was normalized [40]. Additionally, Song et al. calculated the power spectral density (PSD) of the BBI series using the FFT or AR Model, which was denoted as the HRV spectrum of the BCG signal. The HF, the LF and the LF/HF components of the HRV spectrum were used as the features to distinguish AF from NAF [58]. Yu et al. used FFT to extract four frequency features of BCG signals, including spectral entropy, dominant frequency, magnitude and ratio of the dominant frequency [2]. Tadi et al. applied spectral flux and spectral peaks to identify AF using SCG signals [34]. Among them, spectral flux was calculated by comparing the power spectrum of one frame with the power spectrum of the previous frame. Moreover, spectral peaks referred to the amplitudes of the six largest peaks in the density spectrum, which was estimated by Welch’s PSD.

### 3.3. Time-Frequency-Domain Features

Biosignals are highly non-stationary in nature, especially in the presence of arrhythmias [17]. Time-frequency-domain features describe the change in frequency over time, which is beneficial for real-time AF monitoring.

(a) Time-varying PSD/FFT: Time-varying FFT can reduce the edge effect of FFT and smoothen the spectrum. For AF diagnosis, Hurnanen et al. extracted the spectral entropy obtained by the time-varying PSD of the rectified SCG segments with the Hamming window [31,32,33,34,35,36]. Brüser et al. calculated seven time-frequency features based on the PSD of BCG signals to distinguish AF from SR, such as skewness and kurtosis [18,57].

(b) Wavelet transform: Wavelet transform (WT) is an ideal tool for time-frequency analysis and processing, which can inherit and develop the short-time FFT localization and provide a “time-frequency” window to overcome the limitations of fixed window size. Yu et al. analyzed the atrial activity by capturing the power distribution profile using time-invariant stationary WT of BCG signals [2]. Shashikumar et al. extracted the wavelet power spectrum of PPG signals to detect AF [45]. Among them, a large ensemble of surrogate data was generated by Morlet wavelet, and the wavelet power was calculated using the 95-percentile of the power as the threshold.

### 3.4. Nonlinear Features

In nonlinear measurements, a signal can be regarded as a complex system. Moreover, then nonlinear features are commonly used to describe the complexity of the information [58]. There are five nonlinear characteristics adopted in AF diagnosis: approximate entropy (ApEn), turning point ratio (TPR), sample entropy (SamEn), Shannon entropy and Poincaré plot analysis (PPA).

(a) Approximate Entropy: ApEn is a self-similarity parameter that quantifies the unpredictability of fluctuations in a time-series [34]. The larger the ApEn is, the more irregular the signal is. Lahdenoja et al. extracted the ApEn parameter from SCG signals to diagnose AF [33,34,35,36].

(b) Turning–Point Ratios: TPR is a nonparametric statistical approach to determine the signal’s randomness [34]. Lahdenoja et al. calculated TPR to detect AF by defining the operator RD representing the total number of consecutive increases and decreases in the SCG segment [33,34,35,36]. Shan et al. calculated the TPR of PPG signals to identify AF, which is the ratio of the turning point to the total data length. Each beat in the BBI series was compared to its two nearest neighbors, and a turning point is defined if it is greater or less than two neighbors [48,59].

(c) Sample Entropy: SamEn is a nonlinear method, which has been widely used to evaluate the physiological control mechanisms [48]. SamEn is also a modified version of ApEn, which is considered to assess the complexity or dynamics of physiological time-series. For AF diagnosis, Shan et al. and Shashikumar et al. severally extracted the SampEn feature from PPG signals [43,48,53,59].

(d) Shannon entropy: Shannon entropy is a common definition of entropy in information theory. Shannon’s measure of information is the probability of symbols representing the amount of uncertainty or randomness in the data [48]. Shan et al. and Krivoshei et al. severally calculated the Shannon entropy parameter of PPG signals to detect AF [43,48,53,59].

(e) Poincaré plot analysis: The Poincaré plot describes the nonlinear dynamics of a phenomenon that can recognize hidden correlation patterns in a time-series. It consists of a visual representation of the values of each pair of successive elements in a time-series into a simplified phase space or Cartesian plane. At successive times, a series of these points outline a curve, or trajectory, which describes the system’s evolution [60]. For AF diagnosis, Krivoshei et al. and Shi et al. applied the Poincaré plot to analyze the rhythmic variation of PPG signals, respectively [40,53].

### 3.5. Other Features

In addition to the common features mentioned above, some studies defined new features to characterize AF. For example, Wen et al. converted the original BCG signal into an energy signal and defined three concepts of “peak”, “burr”, “trough”. Moreover, then four new data sequences were defined: “peak intervals”, “relative difference of peak amplitude”, “relative trough”, and “burrs between adjacent peaks”. Finally, the mean value, variance, skewness, and kurtosis of these four data sequences were calculated as 16 features [16].

To sum up, the feature extraction methods for AF detection using cardiac dynamics signals are listed in Table 2.

## 4. Classifier

Generally, the extracted features or entire segments are fed into classifiers to automatically diagnose various diseases, including binary or multiclass classification problems [35]. AF detection is usually a binary classification problem, which divides the input vectors into SR or AF. There are three main types of classifiers applied in previous work: machine learning (ML), deep learning (DL), and statistical classifiers.

### 4.1. Machine Learning

ML is a type of supervised learning method that tests and trains labeled data [6,61]. Over the past decades, ML classifiers have been broadly and successfully applied to AF detection for cardiac dynamics signals, including support vector machine (SVM), random forest (RF), k-nearest neighbor (KNN), naïve Bayes (NB), linear least-squares, k-means clustering, boosting, linear discriminant analysis (LDA), quadratic discriminant analysis (QDA), bagged trees (BT), and bootstrap-aggregated decision trees. Among them, SVM and RF were verified as the optimal classifiers, which achieved superior performance in AF detection.

#### 4.1.1. Support Vector Machine

For binary classification problems, SVMs map the input vectors from low-dimensional space to high-dimensional space using kernel functions [49]. The purpose is to find the optimal classification hyperplane, where the maximum margin between two classes is obtained. SVM obtains the kernel function parameters from the training datasets and uses them to classify the testing datasets [62].

In terms of PPG signal, Ferranti et al. first trained SVM to detect AF using PPG signal and used principal component analysis (PCA) and packing methods to reduce the dimensionality of the dataset [49]. The accuracy and sensitivity reached 90% and 96.67%, respectively. Moreover, later, Shan et al. proposed a cost-sensitive SVM to address the class imbalance problem [59]. The PPG signal was selected to classify patients with AF and NAF, and the accuracy was improved to 95.7%. Yang et al. used radial basis function kernel support vector machine (RBF-SVM) to classify PPG signals for AF detection, and the algorithm performed best in classifying 10 s PPG with an accuracy of 90% [57]. Similarly, in Shi et al.’s study, RBF-SVM was also used to classify 30 s PPG signals for AF detection, and finally, the AF detection accuracy improved to 92.56% with a larger amount of data used [40]. In terms of SCG signals, Lahdenoja et al. compared three ML models to classify the 10 s SCG segments, and the kernel support vector machine (KSVM) performed best and achieved an accuracy of 97.4% [33]. Based on Lahdenoja’s work, Iftikhar et al. applied KSVM and RF to classify the SCG segments from a larger dataset, and KSVM outperformed RF, with improved specificity and accuracy of 100% and 98.4%, respectively [35]. In terms of BCG signals, Yu et al. employed three ML models to classify 30 s BCG segments, and the fine Gaussian SVM model achieved the highest accuracy of 92.2% [2]. In the study by Wen et al., both KSVM and SVM were used in the experiments [16]. 60 s BCG segments were applied to identify AF, and the SVM performed the best among the five tested classifiers and achieved sensitivity, precision, and accuracy of 96.8%, 92.8%, and 94.5%, respectively. Brüser et al. trained an SVM to classify 30 s of BCG signals from patients with AF. The result obtained 96.2% sensitivity and 91.9% specificity [17].

#### 4.1.2. Random Forest

RF was applied to physiological signal measurements by Breiman back in 2001, which is usually more efficient and provides more accurate results than the simple decision trees approach. RF is based on a tree structure constructed following bagging and bootstrap methods. The dataset is subdivided into several parts by the bootstrap, and then a decision tree is learned from each part [63].

For AF detection, Eerikäinen et al. used RF with 30 s PPG segments and achieved a sensitivity of 93.6% and a specificity of 88.2% [43]. Koivisto et al. used the RF classifier to classify 5 s BCG signals for detecting atrial fibrillation and compared it with the SVM classifier [22]. RF performed better than SVM, obtaining 100% sensitivity and 93.3% specificity. Brüser et al. used 30 s BCG segments and evaluated seven popular ML algorithms. RF achieved the best performance, with Matthews correlation coefficient, mean sensitivity, and mean specificity of 92.1%, 93.8%, and 98.2%, respectively [18]. Tadi et al. applied three classifiers to detect AF SCG signals and compared six lengths of segments [34]. The RF classifier based on bootstrap aggregation (bagged) decision tree achieved the best performance in classifying 10 s SCG segments, yielding results with 97% accuracy.

Moreover, different segment lengths affect the performance of RF classifiers. In the study by Tadi et al., SCG segments with the length of 10, 20, 30, 40, 50 and 60 s were used to classify AF patients. The RF classifier achieved the highest classification performance with 97% and 95% accuracy and specificity with 10 s SCG signal segments, while a slightly poorer performance was achieved when the segments were 30 s in length, achieving 95% accuracy and 92.7% specificity [34].

#### 4.1.3. Other ML Models

Moreover, several other ML models have been applied to AF detection, such as KNN, boosting, and NB.

KNN is a supervised learning method based on the closest training data. In the classification task, voting is usually used to select the category that appears most in *k* samples as the prediction result [64]. Yu et al. presented a “fine” KNN model with a neighbor number of 1 to classify AF and SR using 30 s BCG signals, and the accuracy reached 91.94% [2].

Boosting is also a supervised learning model consisting of many weak classifiers to form an integrated strong classifier. In integration, the weak classifiers are given different weights according to their classification accuracy, which can reduce the bias in supervised learning. In recent studies, this model has been extended to many sub-algorithms, such as gradient boosting [65], adaBoost (include real adaBoost, gentle adaBoost and modest adaBoost) [66]. Tadi et al. used a noise-tolerant boosting model, an ensemble of classification trees to detect AF. Moreover, the accuracy of this model was 97.6% using 10 s SCG segments [34].

Naïve Bayes classifier works based on Bayes’ theorem. Prior and conditional probabilities are calculated from the dataset [67]. For continuous variables, a univariate Gaussian distribution is used to estimate their class-conditional marginal densities. A histogram is used for discrete attributes. The underlying assumption of this classifier is that predictor attributes are independent; hence, it is called naïve. This simple classifier is popular considering that it performs well for even small-scale data and can be trained in batches while having less computational complexity. In the study by Song et al., the naïve Bayesian classification method was applied to classify AF segments from 18 patients using BCG signals [58]. Moreover, the results provided a classification precision of 92.3%. In addition, linear least-squares, k-means clustering, and extreme gradient boosting can also be used for SCG signals to detect AF [31,32,45].

### 4.2. Deep Learning

DL is a learning process that uses deep neural networks to solve feature expressions [68]. The aim is to build a neural network that can mimic the thinking and judging process of the human brain. Compared with traditional ML models, DL requires a large amount of data for analysis and training to achieve higher accuracy. At present, the main classifiers based on DL are DCNN, end-to-end learning network and CNN, which have been applied to classify PPG and BCG signals.

Ming-Zher Poh et al. proposed a deep convolutional neural network model (DCNN) to detect AF using 17 s PPG segments [51]. This model used features such as peaks, troughs, and upward and downward slopes and achieved an accuracy of 96.1% and a specificity of 99.0%. This study validated the feasibility of applying a DL system to detect AF from raw PPG waveforms.

Aliamiri et al. proposed an end-to-end learning network based on wearable devices to detect AF from PPG signals [47]. 30 s PPG segments were fed to the model directly, and the accuracy of AF detection was improved to 98.19%.

CNN is a general classifier consisting of a series of convolutional layers, which can extract features consecutively to improve the compatibility and accuracy of the model. Shashikumar et al. established a CNN model to identify AF using 30 s PPG segments [45]. The model’s weight was optimized by the root-mean-square prop (RMSProp) algorithm, and the final accuracy was 91.8%. Jiang et al. presented a new method for detecting AF based on CNN [20]. The principle was to train the CNN model with 20,000 ECG segments first and then classified the 2000 BCG segments from 19 subjects with the trained CNN network using the transfer learning method. The final classification accuracy, sensitivity, and specificity were 95.8%, 98.3% and 93.3%, respectively. Later, Jiang et al. utilized an integrated framework with CNN and Bi-LSTM networks based on the attention mechanism to improve the robustness of AF detection [21]. In this work, the 1D morphology feature extracted from the Bi-LSTM network and both the 1 s segments and 24 s segments were selected as input data. Compared with the classical ML classifiers, the performance of the proposed method was superior with 94.7% accuracy, 93.5% specificity, 95.9% sensitivity, and 93.7% precision.

### 4.3. Statistical Analysis

Statistical analysis is the unmasking of patterns and trends in future data by collecting and interpreting known data, which can be used to collect research interpretation, statistical modeling, or to design surveys and studies [69]. For AF detection, the Markov model, logistic regression, and elastic net logistic model are three common statistical classifiers using PPG signals.

Markov model is a statistical model, which assumes that the future state depends only on the current state. Therefore, it is powerful to deal with time-series classification problems. Bonomi et al. employed a 1st order Markov model to estimate the probability of arrhythmias in AF induced by BBI derived from 30 s PPG signals [41]. The sensitivity and specificity were 97 (±2)% and 99 (±3)%, respectively. The results showed that the Markov model could robustly handle the missing beats in the time interval series. G. Bonomi et al. proposed an AF detection algorithm that used the Markov model to classify PPG signals from 20 AF patients [42]. The segment length was set to 60 s, and the accuracy of AF detection was higher than 96%.

Logistic regression is a common statistical analysis method, which can be used for both binary and multiclass classification. When carrying out binary classification problems, logical regression uses the S function to limit the output to two values, 0 and 1. Tang et al. applied a 30 s PPG signal to classify AF and SR using logistic regression [48]. The area under the receiver operating characteristic (ROC) curve was 97.3%.

Zou et al. proposed the elastic net, a new regularization and variable selection method [70]. Real-world data and a simulation study show that the elastic net often outperforms the lasso while enjoying a similar sparsity of representation. In addition, elastic net encourages grouping effects, where strongly correlated predictors tend to enter or exit the model together. The elastic net is particularly useful when the number of predictors is much bigger than the number of observations. Nemati et al. designed an elastic net model to detect AF. In this model, 30 s PPG signals were used, and the result achieved 95% accuracy, 97% sensitivity and 94% specificity [71].

To sum up, classifiers for AF detection using cardiac dynamics signals are listed in Table 3.

## 5. Conclusions

This study sums up the major research on non-invasive AF detection methods based on cardiac dynamics signals, which are more suitable for long-term unobtrusive cardiovascular monitoring at home. In the existing studies, BCG, SCG and PPG signals all have shown good specificity for AF classification, and a series of wearable devices have been designed to diagnose AF in daily life.

For BCG signals, EMFi or PVDF sensors were designed as cushions or mattresses to monitor AF in daily life, especially during sleep. Traditional detection methods extract temporal and frequency features of the preprocessed BCG segments and then apply the ML classifiers to distinguish AF from NAF. However, the features’ characteristics and classification performance depend mainly on the signal quality. Therefore, recent studies have focused on the DL classifiers, which can omit the feature extraction module. The experimental results demonstrate the superiority of DL methods compared to ML methods. However, the accuracy of DL methods is influenced by the amount of data contained in the dataset, which is a challenge for BCG signal processing.

For SCG signals, the MEMS is the main sensor, and smartphones are popular AF detecting devices, which are convenient and widely used. We can place the smartphone on the chest to monitor the arrhythmia anytime and anywhere. Due to the acquisition location close to the heart, the signal quality of SCG is superior to that of BCG. However, BCG is more suitable for long-term monitoring. The common SCG-based AF detection method also extracts the temporal and frequency features of the preprocessed SCG segment and then applies an ML classifier to distinguish AF from NAF. Hence, far, there is no study on AF detection with SCG signals using a DL classifier. The balance between the computational burden of the DL method and the classification performance of the ML method determines its application in smartphones.

For PPG signals, PD sensors are equipped on wearable devices, such as watches to monitor cardiac function. Meanwhile, the camera on the smartphone can also provide PPG signals. Undoubtedly, it is more convenient to collect the signal with the camera, but the preprocessing method is more complicated. In addition, for the PPG waveform computationally reconstructed from the camera, the characteristic components contained in the morphology are weakened, which is usually only effective in extracting HRV parameters but not for diagnosing specific heart diseases. The classical AF detecting method of PPG is to recover the PPG waveform, extract the HRV or nonlinear features, and then feed it into an ML classifier to detect AF. Recently, the DL classifiers and statistical classifiers have been applied to screen AF using PPG signals. The feature extraction module also could be omitted. The classification performance is satisfactory, and porting the algorithm to the mobile phone may become the next task. In addition, it is a challenge to improve the characterization performance of the reconstructed PPG waveform.

Moreover, there are still some common limitations in the existing studies.

First is the scarcity of raw data sources. Because cardiac dynamics signals are generally weak and susceptible to perturbations, a slight movement of the subject will affect the quality of the signal collected, leading to a paucity of labeled data and subjects available. Furthermore, the elderly account for most AF patients, and relatively few data have been recorded from normal subjects, resulting in a severe imbalance for data distribution. The drawbacks constraint the accuracy and generalizability of existing AF detection algorithms.

Moreover, unlike the ECG signal, the morphology of cardiac dynamics signals depends on different devices and different subjects. Therefore, extracting the universal features from different cardiac dynamics signals to characterize AF is a key challenge for AF screening in daily life. Additionally, the waveform mechanism of cardiac dynamics signals is not thoroughly understood, so all experimental labels rely on synchronized ECG signals, limiting the detection conditions and the data sources.

**Figure 5:**
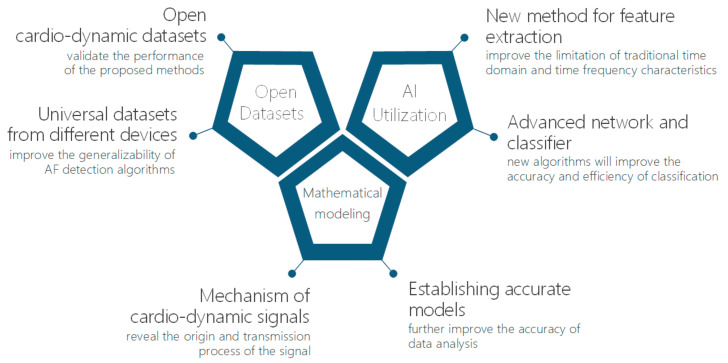
Future work.

As a result, the following studies can be carried out in future work as illustrated in Figure 5.

First, open cardiac dynamics datasets for AF detection are necessary to validate the performance of the proposed methods. Universal datasets from different devices will improve the generalizability of AF detection algorithms and facilitate using cardiac dynamics signal applications in cardiovascular disease diagnosis.

Second, an increasing number of artificial intelligence approaches will be applied to extract the characterization parameters automatically and adaptively. Common temporal and frequency features are not suitable for a wide range of cardiac dynamics signals. Artificial intelligence approaches, such as DL algorithms, have been successfully used to optimize AF classification performance. Therefore, with developing artificial intelligence technology, the detection accuracy will be further improved.

Finally, studying the mechanisms of cardiac dynamics signals will reveal the physiological and dynamic principles of the signal production and transmission processes. Hence, more precise mathematical models will be further established to analyze cardiac function and cardiovascular system performance. Furthermore, the personalized AF or cardiovascular disease monitoring system based on cardiac dynamics signal models would benefit unobtrusive healthcare at home.

## Figures and Tables

**Table 1:** Cardiac dynamics signals acquisition methods for AF detection.

Signal	Instrument/Sensor		Sampling Rate
BCG	EMFi	L-series (290 × 600 mm) of Emfit (Finland) [16]	125 Hz
		EMFi sensor (40 × 79 cm) of Emfit (Finland) [2]	125 Hz
		EMFi sensor (30 × 60 cm) of Emfit (Finland) [17,18,19]	128 Hz
	PVDF	Polyvinylidene fluoride (PVDF) sensor [20,21]	125 Hz
		Murata BCG sensor (SCA10/11H) [22]	125 Hz
	MEMS	LSM6DSM always-on 3D accelerometer and 3D gyroscope [23]	-
SCG	Single MEMS	Digital output three-axis MEMS (Free scale Semiconductor, MMA8451Q) with 14 bits of resolution [30,31]	800 Hz
		Analog output one-axis MEMS accelerometer (VTI Technologies Oy, SCA620) [24]	3000 Hz
		MEMS pressure sensor element (SCB10H) [32]	1000 Hz
	Smartphone	Sony Xperia Z Series Smartphone (a three-axis accelerometer inside the smartphone and the six data channels of three gyroscopes) [33]	200 Hz
		Sony Xperia Z1 or Z5 smartphone (sing a custom-designed Android application) [34,36]	200 Hz
		Smartphone [4,35]	200 Hz
PPG	PD	Philips heart and motion detection module’s (CM3 Generation3, Wearable Sensing Technologies) wrist wearable sensor [41,42,43]	128 Hz
		Earlobe PPG sensor (HeartSensor HRS-07UE, BINAR Integrated Mobile Systems, Washington, DC, USA) [46]	300 Hz
		Smart wristwatch provided by Samsung (“Simband”) [44,45]	128 Hz
		Samsung gear device [47]	100 Hz
		Bedside monitor (IntelliVue MP70, Philips, Netherlands) [48]	128 Hz
		PPG Empatica E4 wristband [49]	64 Hz
	Camera	RGB network camera (Dell Precision M6400, 30 frames per second, resolution 1280 × 720) [50]	200 Hz
		Smartphone (iPhone 4S, Apple, Inc., Cupertino, CA, USA) [51,52,53]	30 Hz
		Samsung Galaxy 6 smartphone and Samsung Galaxy S8 Plus smartphone [54]	-

**Table 2:** Feature extraction methods for AF detection using cardiac dynamics signal.

Feature Type	Features	Signal	Method
Time-domain	Signal morphology	BCG	Skewness and kurtosis [17]
			Skewness, kurtosis, standard deviation, the difference between the maximum and minimum values of each segment [18]
			Entire BCG segment [20]
		SCG	Entire SCG segment [30]
			Variance of the difference between maximum and minimum [24]
			Zero-crossing ratio [34]
		PPG	Entire PPG segment [47,51]
			Kurtosis [43]
	Time interval	SCG	AO–AO interval [24]
		PPG	BBI [41,42]
	Heart rate	BCG	HR from sensor built-in algorithms [22]
		SCG	IHR [34,35]
			HR from the median of the eight BBI [34]
			HR approximation was achieved by computing short segment autocorrelations [36]
		PPG	HR from means of the location of each PPG waveform trough [54]
	HRV	SCG	Means of the median absolute difference of the cardiac cycle durations [31]
			RMSSD and the median difference based on the successive SCG BBI [34]
			Root-mean-square of the successive median absolute difference of SCG BBI and the two higher-order HRV parameters [33,35]
			Median absolute difference of the obtained BBI [36]
		BCG	Mean, standard deviation of BBI and RMSSD [58]
		PPG	Normalized SD and RMSSD [59]
			RMSSD, mean, SD [40]
			SD, a robust version of SD, and a weighted SD [45]
			Avg ∆ SS, SDSS, pNNx, CVSS [46]
			SD [43]
			RMSSD [44,48,53]
Frequency domain	FFT/PSD	SCG	Spectral flux and the spectral peaks [34]
		BCG	Spectral entropy, the dominant frequency, and the magnitude and ratio of the dominant frequency [2]
			HF, LF and the LF/HF components [58]
		PPG	LF, HF and LF/HF [48,59]
			LF, HF and normalized LF/HF [40]
			Spectral entropy [43]
Time frequency domain	Wavelet	BCG	Power distribution profile using time-invariant stationary WT [2]
		PPG	The wavelet power spectrum [45]
	Time-varying PSD/FFT	SCG	Spectral entropy [31,32,33,34,35,36]
		BCG	Seven time-frequency features based on PSD, such as skewness, kurtosis [18,57]
Nonlinear	Approximate entropy estimate (APEN)	SCG	ApEn is a self-similarity parameter that quantifies the unpredictability of fluctuations in a time-series [33,35,36]
	Turning point ratios (TPR)	SCG	Nonparametric statistical approach to determine the randomness of the signal [33,34,35,36]
		PPG	Ratio of the turning point to total data length [48,59]
	Sample entropy (SampEn)	PPG	Modified version of ApEn, which is considered to assess the complexity or dynamics of physiological time-series [43,45,48,59]
	Shannon entropy	PPG	Common entropy definition in information theory [43,48,53,59]
	Poincaré plot analysis (PPA)	PPG	SD1 (axis vertical to the line of identity), SD2 (axis along the line of identity) [40] and SD1/SD2 [53]
Other	New defined	BCG	Mean value, variance, skewness, and kurtosis of four new defined data sequences [16]

**Table 3:** Classifier for AF detection using cardiac dynamics signals.

Classifiers	Models		Signal	Dataset	Performance	Comparison
ML	SVM		BCG	8 h data from 37 subjects [16]	SEN = 96.8% PRE = 92.8% ACC = 94.5%	NB, BAT, RF, DT
				7.5 h data from 12 AF patients [2]	ACC = 92.2% SEN = 95.82%	BT, KNN
				2 h data from 10 AF patients [17]	SEN = 96.2% SP = 91.9%	-
			SCG	16 AF patients, 23 healthy individuals [33]	ACC = 97.4% SP = 100%	KSVM, RF
				3 min data from 23 healthy individuals, 40 AF patients [35]	ACC = 98.4%	RF
			PPG	468 AF patients [59]	ROC = 97.1% SEN = 94.2% ACC = 95.7%	-
				10 min data from 30 AF patients and 31 healthy individuals [49]	ACC = 90% SEN = 96.67%	-
				10 min data from 30 AF patients and 30 healthy individuals [40]	SEN = 91% SP = 94.11% ACC = 92.56%	-
				11 AF patients [57]	ACC = 90%	-
	RF		BCG	30 min BCG data from 20 AF patients and 15 healthy individuals [22]	SEN = 100% SP = 93.3%	SVM
				45 min data from 10 AF patients [18]	Matthews correlation coefficient = 0.921 SEN = 93.8% SP = 98.2%	LDA, QDA, SVM, NB, BoT, BAT
			SCG	3 min data from 435 subjects, including 190 AF patients and 245 healthy individuals [34]	AUC = 0.972~0.983	KSVM
			PPG	24 h data from 40 subjects (14 with AF) [43]	SEN = 93.6% SP = 88.2%	-
	Others	NB	BCG	18 subjects [58]	PRE = 92.3% ACC = 92.30%	-
		Linear least-squares	SCG	119 min of AF data 126 min of SR data from 13 patients [31]	TPR = 99.9% TNR = 96.4%	-
		K-means clustering		10 min data from 7 AF patients [32]	SEN = 99.1% PRE = 100%	-
		Extreme gradient boosting		three minutes data from 150 AF patients and 150 healthy individuals [36]	AUC = 0.98	LR, RF
		K-nearest neighbor	PPG	11 AF patients [57]	ACC = 90%	KSVM
DL	CNN		BCG	8 h data from 19 patients [20]	ACC = 95.8% SEN = 98.3% SP = 93.3% PRE = 93.7%	-
				8 h data from AF patients [21]	ACC = 94.7% SP = 93.5% SEN = 95.9% PRE = 93.7%	-
			PPG	5 min data from 45 AF patients and 53 healthy individuals [45]	AUC = 0.95 ACC = 91.8%	-
	End to end model		PPG	19 AF patients [47]	ACC = 98.19%	-
	DCNN		PPG	17 s PPG waveforms, 149,048 PPG waveforms from 3039 subjects [51]	SEN = 95.2% CI = 88.3%~98.7% SP = 99.0% ACC = 96.1%	-
Statistical analysis	Markov model		PPG	16 AF patients and 11 healthy individuals [41]	SEN = 97 ± 2% SP = 99% ACC = 98%	-
				24 h data from 20 AF patients [42]	SEN = 97% SEN = 93% SP = 100% ACC > 96%	-
	Logic regression		PPG	1, 2, and 10 min of data from 666 AF patients [48]	AUC = 97.2% SEN = 94.0% ACC = 96.2%	-
	Elastic net logistic model		PPG	3.5 to 8.5 min data from 15 AF patients and 31 healthy individuals [71]	Acc = 95% Sen = 97% Sp = 94% AUC = 99%	-

## Data Availability

The study did not report any data.
